# Analysing the Expression of Eight Clock Genes in Five Tissues From Fasting and Fed Sows

**DOI:** 10.3389/fgene.2018.00475

**Published:** 2018-10-18

**Authors:** Tainã Figueiredo Cardoso, Raquel Quintanilla, Anna Castelló, Emilio Mármol-Sánchez, Maria Ballester, Jordi Jordana, Marcel Amills

**Affiliations:** ^1^Department of Animal Genetics, Centre for Research in Agricultural Genomics (CRAG), CSIC-IRTA-UAB-UB, Campus de la Universitat Autònoma de Barcelona, Bellaterra, Spain; ^2^CAPES Foundation, Ministry of Education of Brazil, Brasilia, Brazil; ^3^Animal Breeding and Genetics Programme, The Institute for Research and Technology in Food and Agriculture (IRTA), Barcelona, Spain; ^4^Departament de Ciència Animal i dels Aliments, Universitat Autònoma de Barcelona, Bellaterra, Spain

**Keywords:** clock genes, food ingestion, nutrition, RT-qPCR, pig

## Abstract

In a previous study, we observed that circadian clock genes are differentially expressed in the skeletal muscle of fasting and fed sows. The goal of the current work was to investigate if these genes are also differentially expressed in tissues containing the central (hypothalamus) and peripheral (duodenum, dorsal fat, muscle, and liver) clocks. As animal material, we used 12 sows that fasted 12 h before slaughtering (T0) and 12 sows that were fed *ad libitum* 7 h prior slaughtering (T2). Tissue samples were collected immediately after slaughter and total RNA was subsequently extracted. The expression of the *ARNTL, BHLHE40, CRY2, NPAS2, NR1D1, PER1, PER2*, and *SIK1* genes was measured by quantitative reverse transcription PCR. The numbers of clock genes showing differential expression before and after feeding varied depending on the tissue i.e., four in dorsal fat and duodenum, six in skeletal muscle, and seven in the liver. In contrast, none of the eight analysed genes displayed a significant differential expression in hypothalamus, the tissue where the central clock resides. This result supports that the differential expression of clock genes in the four tissues mentioned above is probably induced by nutrition and not by the central clock entrained by light. Moreover, we have observed that the *NPAS2* and *ARNTL* genes display positive log_2_(FC) values in the five tissues under analysis, whilst the *CRY2, PER1* (except dorsal fat) and *PER2* (except hypothalamus) genes generally show negative log_2_(FC) values. Such result might be explained by the existence of a negative feedback loop between the *ARNTL/NPAS2* and *CRY/PER* genes. Collectively, these results support that nutrition plays an important role in modulating the timing of porcine peripheral circadian clocks. Such regulation could be essential for coordinating the subsequent metabolic response to nutrient supply.

## Introduction

Circadian clocks are highly conserved endogenous oscillators controlling a wide repertoire of physiological events, including metabolism and behaviour (Bellet and Sassone-Corsi, 2010). At the molecular level, the rhythmicity of circadian clocks is modulated by several transcriptional feedback loops composed of positive and negative regulators (Eckel-Mahan and Sassone-Corsi, 2013; Partch et al., 2014). The aryl hydrocarbon receptor nuclear translocator-like (ARNTL) transcription factor heterodimerises with either the clock circadian regulator (CLOCK) or its paralogue, the neuronal PAS domain protein 2 (NPAS2), thus activating the expression of the period (*PER*) and cryptochrome (*CRY*) genes. Upon reaching a critical concentration threshold, PER and CRY translocate to the nucleus where they inhibit the activity of the (CLOCK/NPAS2):ARNTL heterodimer, thus establishing a negative feedback loop (Peek et al., 2012; Menet et al., 2014). Cyclical oscillations in the expression of (CLOCK/NPAS2):ARNTL and *PER:CRY* genes modulated by this and other feedback loops promote the establishment of circadian patterns modulating the transcriptional activity of thousands of genes (Partch et al., 2014; Takahashi, 2017). This core mechanism is further refined by the action of additional genes, such as nuclear receptor subfamily 1 group D member 1 (*NR1D1*), basic helix-loop-helix family member E40 (*BHLHE40*), and salt-inducible kinase 1 (*SIK1*) which cooperate to finely tune the rhythmicity of mammalian circadian clocks (Honma et al., 2002; Bugge et al., 2012; Oike et al., 2014).

In mammals, the central circadian clock is located in the hypothalamus, and more specifically in the suprachiasmatic nucleus (Partch et al., 2014). This central clock is fundamentally entrained by the light/dark cycle (Partch et al., 2014). Circadian clocks are also present in peripheral tissues, but they are mainly entrained by feeding/fasting cycles, glucose metabolism, insulin secretion, and temperature (Hastings et al., 2008; Richards and Gumz, 2012; Oike et al., 2014). Circadian clocks have a profound effect on metabolism and gene expression. For instance, a third of the genes in the mouse genome show circadian patterns of expression (Gooley and Chua, 2014). The knockout of specific circadian genes in mice is associated with a broad variety of abnormal metabolic phenotypes including obesity, hyperglycemia, hepatic steatosis, hypertriglyceridemia, hypotriglyceridemia, glucose intolerance, hypoinsulinemia, and cholesterolemia (Gooley and Chua, 2014). The analysis of the murine skeletal muscle transcriptome has shown that genes related with fatty acid uptake and β-oxidation peak in the inactive phase, whilst genes related with carbohydrate catabolism, carbohydrate storage and lipogenesis peak in the early, middle and late active phases, respectively (Hodge et al., 2015).

Whereas circadian clocks have been intensively studied in humans and mice, little is known about the mechanisms by which these clocks respond to external stimuli in domestic species. Zhou et al. (2017) showed that long-chain polyunsaturated fatty acid levels in plasma and liver as well as the hepatic mRNA levels of lipid genes (i.e., *FADS1, FADS2, ELOVL2*, and *ELOVL5*) exhibit diurnal rhythms in pigs. Recently, we compared the patterns of skeletal muscle expression of sows that fasted 12 h before slaughtering (T0) vs. sows that were fed 5 h (T1) and 7 h (T2) before slaughtering (Cardoso et al., 2017). Our results demonstrated the existence of differential mRNA expression of circadian clock genes in the pig skeletal muscle, and such differences were particularly significant in the T0 vs. T2 comparison (Cardoso et al., 2017). However, T0 sows were sampled at 7.30 in the morning and, in contrast, T2 sows were sampled 7 h later, so the changes of circadian clock gene expression could be also due to variations in the amount of light associated with the passing of time. The main goal of the current work was to investigate if circadian clock genes are differentially expressed in the hypothalamus of fasted T0 vs. fed T2 sows as well as in several tissues containing peripheral clocks with a key metabolic role (liver, duodenum, muscle, and dorsal fat).

## Materials and Methods

### RNA Isolation

The experiment was carried out with a group of 24 sows belonging to a commercial Duroc line and born in the same week (January 25th–31st, 2015). After weaning (age = 3–4 weeks), this pig population was transferred to the IRTA-Pig Experimental Farm at Monells (Girona, Spain). All animals were kept under the same feeding and management conditions. Additional details about how these sows were bred and fed can be found in Cardoso et al. (2017). Sample tissues were retrieved from 12 sows that fasted 12 h before slaughtering (T0) and 12 sows that were fed *ad libitum* 7 h prior slaughtering (T2). Tissue samples (liver, dorsal fat, *gluteus medius* muscle, duodenum, and hypothalamus) were collected immediately after slaughter, submerged in RNAlater (Ambion, Austin, TX, United States), and stored at -80°C until RNA extraction. Muscle tissue samples were individually pulverised using a pre-chilled mortar and a pestle. Powdered samples were homogenised in 1 ml TRIzol Reagent (Invitrogen Corp., Carlsbad, CA, United States). Liver, dorsal fat, duodenum, and hypothalamus tissues were directly homogenised in TRIzol Reagent (1 ml). All samples were homogenised with a polytron device (IKA, Denmark). Total RNA was extracted according to the protocol recommended by Chomczynski and Sacchi (1987). In brief, homogenates were centrifuged and visible fat and cell debris were removed. Chloroform (200 μl) was added and samples were centrifuged to separate the nucleic acid and protein phases. Total RNA was precipitated using 500 μl isopropanol and washed with ethanol (75%). Finally, RNA was resuspended with RNase-free water and stored at -80°C. RNA concentration and purity were measured using a NanoDrop ND-1000 spectrophotometer (NanoDrop Technologies, Wilmington, United States).

### Synthesis of Complementary DNA

Complementary DNA synthesis was carried out with the High-Capacity cDNA Reverse Transcription Kit (Applied Biosystems, Foster City, CA, United States) by using 10 μl (100 ng/μl) of total RNA as template in a final reaction volume of 20 μl. One microliter of MultiScribe Reverse Transcriptase (50 U/μl), 2 μl of 10× random primers, 2 μl of 10× buffer, 0.8 μl of 25× dNTP Mix (100 mM) and 4.2 μl of water were added to the reaction. Tubes were incubated for 10 min at 25°C, 2 h at 37°C and 5 min at 85°C to inactivate the reverse transcriptase according to the manufacturer instructions (Applied Biosystems, Foster City, CA, United States). A negative control was made for each tissue with no reverse transcriptase added (-RT control). Complementary DNAs were stored at -80°C until use.

### Primer Design

The eight genes included in this study were selected based on previous results obtained by Cardoso et al. (2017) as well as by performing a literature search (Tahara and Shibata, 2013; Gnocchi et al., 2015). Primers spanning exon-exon boundaries, or alternatively binding at different exons (in order to avoid the amplification of residual contaminating genomic DNA) were designed with the Primer3 software (Untergasser et al., 2012). Primers employed in the amplification of the β-actin (*ACTB*), TATA-Box Binding Protein (*TBP*), and hypoxanthine phosphoribosyltransferase 1 (*HPRT1*) were reported by Ballester et al. (2017). Primer sequences are available in **Supplementary File 1**.

### RT-qPCR

The quantification of mRNA expression by quantitative reverse transcription PCR (RT-qPCR) was performed by using the QuantStudio 12K Flex Real-Time PCR System (Applied Biosystems, Foster City, CA, United States). Four genes i.e., *ACTB, TBP, HPRT1*, and β_2_-microglobulin (*B2M*) were used as endogenous controls (**Supplementary File 2**). Standard curves with serial dilutions from a pool of cDNA from each tissue were made to evaluate the performance of our RT-qPCR assays. Standard curves were used to calculate the efficiency of amplification reactions. Ideally, the efficiency of a PCR should be 100%, implying that in each cycle the amount of target DNA should be duplicated. However, we accepted efficiencies between 90 and 110%, which is common practise. In short, 3.75 μl of cDNA (1/25 dilution), 7.5 μl of SYBR Select Master Mix (Applied Biosystems, Foster City, CA, United States), and 300 nM of each primer were mixed in a final volume of 15 μl. All reactions were done in triplicate. The thermal profile was 10 min at 95°C and 40 cycles of 15 s at 95°C and 1 min at 60°C. A melting curve step (95°C for 15 s, 60°C for 1 min and a gradual increase in temperature with a ramp rate of 0.05°C/s up to 95°C and a final step of 95°C for 15 s) was carried out to confirm the specificity of the assays.

### Data Analysis

Clock gene expression data were normalised taking as a reference the mRNA levels of four reference genes (*ACTB, TBP, HPRT1*, and *B2M*), according to the stability of the gene expression for each assay. Genes selected as reference controls can be found in **Supplementary File 2**. Relative quantification of gene expression differences between T0 and T2 for each tissue was calculated with the 2^-ΔΔCT^ method (Livak and Schmittgen, 2001) by using the following formulae:

ΔΔC_T_ = ΔC_T(T2)_–ΔC_T(T0)_ (calibrator)

ΔC_T(T2)_ = (C_T__target gene__T2_–C_T reference gene T2_) averaged across all T2 samples in each tissue

ΔC_T(T0)_ = (C_T target gene T0_–C_T reference gene T0_) averaged across all T0 samples in each tissue

Data were evaluated with the RT-qPCR data analysis software available in the Thermo Fisher Cloud (Thermo Fisher Scientific, Barcelona, Spain). The statistical significance of the mRNA expression differences between T0 and T2 was assessed with a Student *t*-test and fold-change (FC) was expressed on a logarithmic scale (log_2_). Correction for multiple testing was implemented with the method reported by Benjamini and Hochberg (1995). We considered that gene expression between T0 and T2 was significantly different when two conditions were met i.e., |log_2_FC| > 0.58 and *q*-value< 0.05. All figures were made with the R software^1^. The raw data used in the current work can be found in Figshare (Cardoso et al., 2018).

## Results

**FIGURE 1:**
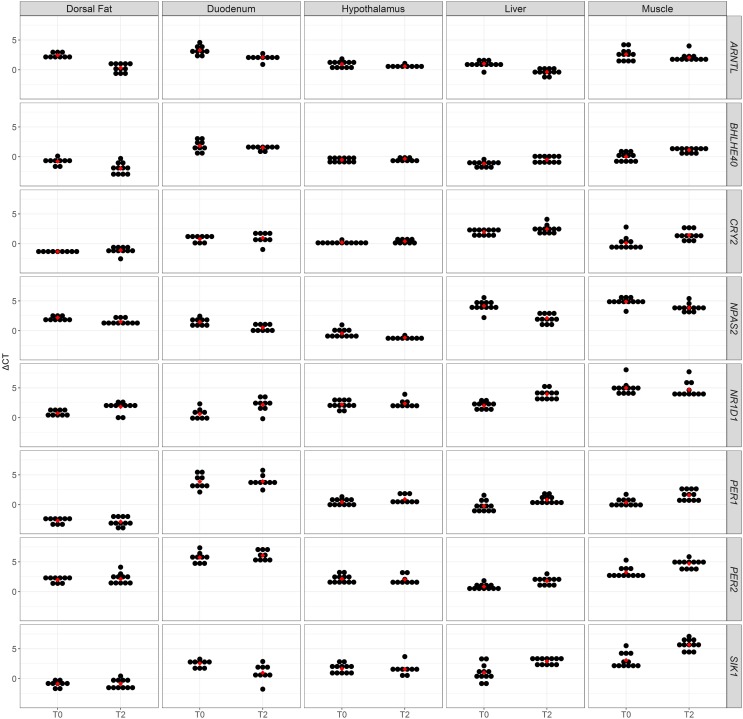
A dot plot of ΔC_T_ values of eight clock genes obtained in five tissues from fasting (T0) and fed (T2) sows. Average ΔC_T_ values are represented as red dots.

**FIGURE 2:**
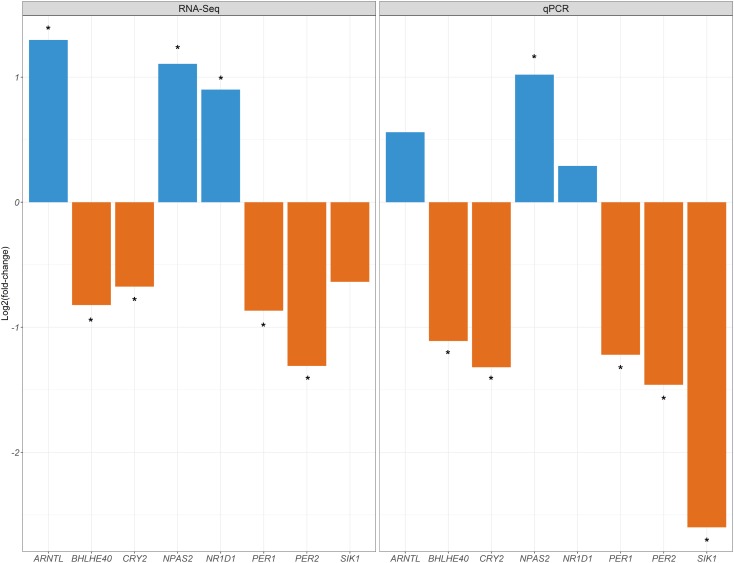
Comparison of the log_2_FC values generated in the current experiment for the muscle tissue (T0 vs. T2) with the log_2_FC values reported by Cardoso et al. (2017) for the same muscle samples (T0 and T2) and genes based on RNA-Seq data. Upregulation and downregulation of mRNA expression are indicated with blue and orange bars, respectively. Asterisks indicate the existence of significant differences in mRNA expression (q-value < 0.05 and | log_2_FC| > 0.58).

We have examined how the expression of eight clock genes changes in fasting (T0) vs. fed (T2 sows). In this study, we did not analyse the *CLOCK* gene because it was not annotated in the *Sus scrofa* genome (*Sscrofa 10.2* assembly^2^). The comparison of the patterns of expression before and after feeding (T0 vs. T2 comparison) indicates that the expression of four (dorsal fat and duodenum), six (skeletal muscle) and seven (liver) genes integrated into or modulating peripheral clocks differs between fasting and fed sows (**Table 1** and **Figure 1**). In contrast, none of the eight analysed genes shows significant variations of expression in hypothalamus, the tissue where the central clock resides (**Table 1** and **Figure 1**). Another interesting observation is that in the four tissues containing peripheral clocks, the sets of genes showing a significant DE are not the same. For instance, in duodenum and muscle, there are four and six genes displaying DE between T0 and T2, but only two of them (*NPAS2* and *SIK1*) are shared by both tissues (**Table 1**). In contrast, the comparison of genes displaying DE in muscle (six genes) and liver (seven genes) demonstrates the existence of a much higher level of overlap i.e., five genes (*BHLHE40, NPAS2, PER1, PER2*, and *SIK1*) are shared by both tissues (**Table 1**). The consistency of our results has been assessed by comparing the log_2_FC values generated in the current experiment for the muscle tissue (T0 vs. T2) with the log_2_FC values reported by Cardoso et al. (2017) for the same muscle samples (T0 and T2) and genes based on RNA-Seq data (**Figure 2**). It can be appreciated that the direction of the observed changes in gene expression are very consistent in both data sets, though there are variations in the magnitude of the change and significance, likely because we are comparing expression data obtained with two different approaches.

**Table 1:** Differential clock gene expression at fasting (T0) and 7 h after eating (T2) in five porcine tissues^1^.

	Dorsal Fat		Duodenum		Hypothalamus		Liver		Muscle	
					
	*q*-value	log_2_FC	*q*-value	log_2_FC	*q*-value	log_2_FC	*q*-value	log_2_FC	*q*-value	log_2_FC
*ARNTL*	**1.20E-05**	**2.12**	**2.00E-02**	**1.16**	4.10E-01	0.36	**3.20E-05**	**1.40**	2.70E-01	0.56
*BHLHE40*	**2.00E-02**	**1.18**	1.00E+00	0.55	7.50E-01	-0.17	**1.00E-02**	**-0.94**	**3.00E-03**	**-1.11**
*CRY2*	7.40E-01	-0.24	1.00E+00	-0.41	7.20E-01	-0.19	1.70E-01	-0.47	**1.00E-02**	**-1.32**
*NPAS2*	**2.00E-02**	**0.68**	**2.00E-02**	**1.05**	1.20E-01	0.71	**2.70E-05**	**2.07**	**4.00E-03**	**1.02**
*NR1D1*	**2.00E-02**	**-1.08**	**3.00E-02**	**-1.48**	1.00E+00	-0.15	**2.40E-05**	**-1.93**	1.00E+00	0.29
*PER1*	1.00E+00	0.16	1.00E+00	-0.05	5.00E-01	-0.48	**1.00E-02**	**-1.05**	**2.00E-03**	**-1.22**
*PER2*	1.00E+00	-0.14	1.00E+00	-0.48	1.00E+00	0.10	**3.00E-02**	**-1.06**	**1.00E-03**	**-1.46**
*SIK1*	1.00E+00	0.00	**4.00E-02**	**1.57**	1.00E+00	0.10	**3.00E-03**	**-1.84**	**1.50E-04**	**-2.60**


*^*1*^Genes showing a significant DE are indicated in bold. A positive log_*2*_FC indicates up-regulation in the T2 treatment and a negative log_2_FC indicates down-regulation in the T2 treatment.*

About the direction (positive = increased mRNA levels in T2, or negative = decreased mRNA levels in T2) of the change in expression before and after feeding, there are genes that show consistent patterns across tissues whilst others do not. For instance, the *NPAS2* and *ARNTL* genes display positive log_2_FC values in the five tissues under analysis (**Table 1**), whilst *CRY2, PER1* (except dorsal fat) and *PER2* (except hypothalamus) generally show negative log_2_FC values (**Table 1**). In contrast, the direction of the expression changes for the *BHLHE40* and *SIK1* genes is quite variable depending on the tissue under consideration. For instance, mRNA levels of the *BHLHE40* gene are decreased in liver and muscle (log_2_FC = -0.94, *q*-value = 1.00E-02 and log_2_FC = -1.11, *q*-value = 3.00E-03, respectively; **Table 1**), but they are increased in dorsal fat (log_2_FC = 1.18, *q*-value = 2.00E-02, **Table 1**). The expression of the *SIK1* gene also presents some degree of tissue specificity, decreasing its mRNA levels in liver and muscle (log_2_FC = -1.84, *q*-value = 3.00E-03, and log_2_FC = -2.60, *q*-value = 1.50E-04, respectively; **Table 1**), but increasing in duodenum (log_2_FC = 1.57, *q*-value = 4.00E-02; **Table 1**).

## Discussion

Clock genes play an essential role in the regulation of metabolic genes in order to coordinate their expression across tissues and organs (Patel et al., 2016; Ribas-Latre and Eckel-Mahan, 2016; Jagannath et al., 2017). We have measured the mRNA expression of eight genes that modulate circadian rhythms in fasting and fed sows. Seven hours after feeding, the expression of four (duodenum and dorsal fat) to seven (liver) clock genes changed in tissues regulated by peripheral clocks (**Table 1** and **Figure 1**). In strong contrast, the hypothalamus, which contains the suprachiasmatic nucleus where the central clock resides, did not show any significant change in the mRNA levels of the eight analysed genes (**Table 1** and **Figure 1**). The T0 and T2 samples were not obtained at the same hour of the day (T2 samples were obtained 7 h later than T0 samples), so the changes of expression that we have observed could be due to the combined effects of nutrition and the passing of time, since both are known to induce oscillations in circadian rhythms. However, it is reasonable to infer that nutrition is the main factor explaining the differences in gene expression that we detect when comparing the T0 and T2 treatments. First of all, it is well known that food is the main entraining cue (*zeitgeber*) of peripheral clocks (Ribas-Latre and Eckel-Mahan, 2016). Many studies suggest that feeding conditions can modify the phase of circadian gene expression in peripheral tissues while leaving the phase of cyclic gene expression in the suprachiasmatic nucleus unaffected (Damiola et al., 2000; Hirota and Fukada, 2004). Indeed, the central clock in the suprachiasmatic nucleus is entrained by the 24 h light-dark cycle and not by feeding. Second, the lack of mRNA expression changes in the hypothalamus that we have observed when comparing RT-qPCR data from T0 and T2 sows suggests that the expression changes that we have observed in the liver, fat, muscle, and intestine are probably not induced by the central clock i.e., they are not mainly due to variations in the amount of light between the T0 and T2 sampling times, but, more likely, to nutrition. We acknowledge, however, that a more definitive conclusion could be reached by collecting T0 and T2 tissue samples at the same hour of the day. Moreover, it would be also necessary to specifically characterise the mRNA expression pattern of the suprachiasmatic nucleus because the hypothalamus is a very complex tissue containing different anatomical areas with specialised functions.

Although we cannot compare mRNA expression across tissues on a quantitative basis because different calibrators have been used in each tissue, we have observed that different sets of the eight genes under study display changes in their mRNA levels depending on the tissue under consideration (**Table 1**), and sometimes the direction of these changes varies across tissues (at some instances the same gene can be downregulated or upregulated depending on the tissue). These observations might be due to the fact that the timing of nutrient absorption varies from tissue to tissue. After food ingestion, the majority of nutrients are absorbed in the intestine and the first organ that glucose and amino acids reach, through the portal system which drains blood from the gastrointestinal tract and other organs, is the liver (Frayn, 2010). Afterward, glucose and amino acids reach the general circulation and they are absorbed in the skeletal muscle and adipose tissue (Frayn, 2010). On the other hand, the absorption and distribution of lipids are delayed, if compared with that of soluble nutrients, because they are packaged as chylomicrons in the intestine (Frayn, 2010). Thus, the expression of clock genes in distinct tissues may reflect to some extent the specific timing of nutrient absorption in each organ. Paradoxically, and despite the sequence of events outlined above, the overlap between the sets of DE genes is much higher in the liver vs. muscle comparison than in the dorsal fat vs. muscle comparison. This apparent contradiction might be explained by additional factors related to tissue function and environmental cues operating at a tissue-specific level. For instance, one fundamental difference between skeletal muscle and adipose depots is that the latter not only absorb nutrients but also release non-esterified fatty acids that are used as a source of energy during fasting (Frayn, 2010). The rhythmic release of free fatty acids and glycerol from adipocytes is locally regulated by clock genes (Shostak et al., 2013; Yoshino and Klein, 2013). In the case of the intestine, an additional key regulatory factor that modulates circadian rhythms is the microbiome. In this regard, Mukherji et al. (2013) have shown that the absence of intestinal microbiota alters drastically circadian gene expression and the cyclic production of corticosterone by the ileum, causing hyperglycemia, hypertriglyceridemia, and insulin resistance (Henao-Mejia et al., 2013; Mukherji et al., 2013). Additionally, specific microbial metabolites, as short-chain fatty acids, may directly modulate circadian clock gene promoting diet-induced obesity by modification of the central and hepatic circadian rhythms (Leone et al., 2015). Another distinctive feature of the gastrointestinal tract is the secretion of large amounts of extrapineal melatonin, an hormone that can contribute to the synchronisation of the peripheral clocks (Liu et al., 1997). Finally, the timing and phasing of clock gene expression differ across tissues because they are subject to distinct regulatory cues and, moreover, they serve distinct metabolic roles. Such organ-specific differences were recently reported in a study analysing the expression of clock genes in mouse liver and stomach and demonstrating that the acrophase of several clock genes was delayed in the stomach (Mazzoccoli et al., 2012). Storch et al. (2002) also showed that the distributions of circadian phases in the liver and heart are substantially different and that a reduced number of genes show circadian regulation in both tissues. Importantly, Storch et al. (2002), highlighted that this specificity of circadian regulation is not explained by the tissue-specific patterns of gene expression.

When comparing the mRNA expression patterns of T0 and T2 sows, we have observed that the *NPAS2* and *ARNTL* genes display positive log_2_FC values in the five tissues under analysis (**Table 1**), whilst *CRY2, PER1* (except dorsal fat) and *PER2* (except hypothalamus) generally show negative log_2_FC values (**Table 1**). These observations could be explained by the existence of a negative feedback loop regulating the expression of the *NPAS2/ARNTL* and *CRY* and *PER* genes. In this way, the NPAS2/ARNTL heterodimers stimulate the transcription of *PER* and *CRY* genes. When PER and CRY reach a certain concentration threshold in the cytosol, they translocate to the nucleus and repress the expression of the *NPAS2/ARNTL* genes (Partch et al., 2014). In consequence, a certain degree of antagonism in the expression of *NPAS2/ARNTL* and *PER/CRY* genes could be anticipated. With regard to NR1D1 (also known as REV-ERBα), it is known that this nuclear receptor binds ROR-specific response elements in the promoter of the *ARNTL* gene, thus hindering the binding of the positive transcription regulator RORα (Nakashima et al., 2008; Mazzoccoli et al., 2012). This inhibitory role of NR1D1 on *ARNTL* mRNA expression agrees well with the fact that most of log_2_FC values of this gene are negative (except in the skeletal muscle).

The *BHLHE40* gene displays positive log_2_FC values in dorsal fat and duodenum, and negative values in hypothalamus, liver, and muscle (**Table 1**). On the other hand, the *SIK1* gene displays a positive log_2_FC value in duodenum and negative values in liver and muscle (**Table 1**), a pattern of expression that resembles that of *BHLHE40*. Interestingly, the functional analysis of the *SIK1* gene has shown that it is expressed in the suprachiasmatic nucleus and that it modulates the entrainment of the central circadian clock by light (Jagannath et al., 2013). Our results indicate that *SIK1* mRNA levels in liver, muscle, and duodenum are also influenced by nutrition, thus suggesting that *SIK1* could also play a role in the fine tuning of peripheral clocks. The general picture that emerges from our results is that the sign of the log_2_FC values of *BHLHE40* and *SIK1* can be positive or negative depending on the tissue under consideration (**Table 1**). Indeed, these genes are not only involved in the maintenance of circadian rhythms but also in many other biological processes, so their mRNA levels are determined by a multiplicity of factors and complex interactions. For instance, BHLHE40 proteins repress the NPAS2/ARNTL transactivation of the *PER1* gene promoter by competing for E-box binding and interacting with ARNTL (Honma et al., 2002). In addition, this transcription factor regulates cell proliferation and differentiation (Shen et al., 1997), adipogenesis (Ozaki et al., 2012), cytokine production by T cells (Lin et al., 2014), apoptosis (Qian et al., 2012) and cellular senescence (Qian et al., 2008). Similarly, the *SIK1* gene performs a broad variety of functions related with inflammation (Lombardi et al., 2016), steroidogenesis (Hu et al., 2015), renal function (Taub et al., 2015), vascular remodelling (Bertorello et al., 2015) and glucose metabolism (Patel et al., 2014), to mention a few, so its biological role goes far beyond the modulation of circadian rhythms and this is reflected in its variable pattern of expression across porcine tissues.

In a previous work, we demonstrated, by using an RNA-Seq approach, the existence of changes in the expression of clock genes in the porcine skeletal muscle of fasting and fed sows (Cardoso et al., 2017). Here, we have confirmed this result by using an RT-qPCR approach and we have also provided evidence that the expression of clock genes experiences changes in three additional tissues regulated by peripheral clocks (duodenum, liver, and dorsal fat) when fasting and fed sows are compared. However, we do not observe such changes in the hypothalamus, which contains the central master clock entrained by light. As a whole, our results are consistent with a scenario where food intake acts as a dominant “timer” to porcine peripheral clocks. Further experiments will be needed to assess the precise role of nutrition as a regulatory factor for clock gene expression in pigs.

## Ethics Statement

Animal care, management procedures and blood sampling were performed following national guidelines for the Good Experimental Practises and they were approved by the Ethical Committee of the Institut de Recerca i Tecnologia Agroalimentàries (IRTA).

## Author Contributions

MA, RQ, and JJ designed the experiments. RQ was responsible for the experimental protocols and generation of animal material. TFC and EMS performed RNA extractions. AC designed the RT-qPCR experiments. TFC carried out the RT-qPCR experiments. TFC and AC analysed the RT-qPCR data. MB contributed to the biological interpretation of the expression data. MA and TFC wrote the paper. All authors contributed to the obtaining of biological samples and read and approved the manuscript.

## Conflict of Interest Statement

The authors declare that the research was conducted in the absence of any commercial or financial relationships that could be construed as a potential conflict of interest.

## Footnotes

1 https://www.r-project.org/

2 https://www.ensembl.org
